# The Effects of Dietary Nitrate Supplementation on Explosive Exercise Performance: A Systematic Review

**DOI:** 10.3390/ijerph19020762

**Published:** 2022-01-11

**Authors:** Rachel Tan, Leire Cano, Ángel Lago-Rodríguez, Raúl Domínguez

**Affiliations:** 1 Faculty of Sports Medicine, Natural Sciences Division, Pepperdine University, Malibu, CA 90263, USA; rachel.tan@pepperdine.edu; 2 Independent Researcher, 48991 Getxo, Spain; leire_140@hotmail.com; 3 Movement, Brain and Health Group, Center of Higher Education Alberta Giménez, 07013 Palma de Mallorca, Spain; 4 Departamento de Motricidad Humana y Rendimiento, Universidad de Sevilla, 41013 Sevilla, Spain; rdherrera@us.es; 5 Studies Research Group in Neuromuscular Responses (GEPREN), University of Lavras, Lavras 37200-000, Brazil

**Keywords:** beetroot juice, ergogenic aid, power, sports nutrition, muscle, nitric oxide

## Abstract

Dietary nitrate supplementation is evidenced to induce physiological effects on skeletal muscle function in fast-twitch muscle fibers and may enhance high-intensity exercise performance. An important component of sport-specific skills is the ability to perform explosive movements; however, it is unclear if nitrate supplementation can impact explosive efforts. We examined the existing evidence to determine whether nitrate supplementation improves explosive efforts lasting ≤ 6 s. PubMed, Scopus and Directory of Open Access Journals (DOAJ) were searched for articles using the following search strategy: (nitrate OR nitrite OR beetroot) AND (supplement OR supplementation) AND (explosive OR power OR high intensity OR high-intensity OR sprint* OR “athletic performance”). Out of 810 studies, 18 were eligible according to inclusion criteria. Results showed that 4 of the 10 sprint-type studies observed improved sprint time, power output, and total work in cycling or running, whereas 4 of the 10 resistance-based exercise studies observed improvements to power and velocity of free-weight bench press as well as isokinetic knee extension and flexion at certain angular velocities. These results suggest that nitrate potentially improves explosive exercise performance, but further work is required to clarify the factors influencing the efficacy of nitrate in different exercise modalities.

## 1. Introduction

The ability to generate power (i.e., the product of force and velocity) during explosive movements is a key determinant for performance in various sports [1]. Explosive movements can be characterized by efforts that last ≤ 6 s as the main energetic contribution is derived from the phosphocreatine system [2]. Maximal power production is linked to factors such as the rate of force development (i.e., velocity of contraction) [3], strength [4], and the recruitment of type II muscle fibers [5], and thus the manipulation of such variables, through exercise or dietary interventions, may improve explosive efforts [6,7].

The International Olympic Committee (IOC) recently classified five supplements to have a strong evidence base for enhancing high-intensity exercise performance, which includes caffeine, beta-alanine, sodium bicarbonate, beetroot juice, and creatine [8]. Out of these aforementioned supplements, dietary nitrate (NO_3_^−^), administered most often as concentrated beetroot juice, is the only supplement from a natural food source [9], and the notion that this simple, vegetable-derived strategy can improve athletic performance has intrigued scientists and athletes alike [10]. Dietary NO_3_^−^ can be obtained from the diet through consuming green leafy vegetables, which have ~4 mmol of NO_3_^−^ per 100 g of produce [9]; however, beetroot juice supplements (one to two doses of concentrated shots, each containing 70 mL × ~6.4 mmol of NO_3_^−^) would be more practical compared to ingesting nitrate-rich meals before competition or training to minimize gastrointestinal discomfort. To date, based on the available evidence, a minimum dose of at least ~6 mmol of NO_3_^−^, ingested within 2–3 h prior to the start of exercise, is required to induce physiological effects [11]. Although high amounts of NO_3_^−^ have been traditionally viewed to have detrimental physiological effects, diets rich in NO_3_^−^ content from fruits and vegetables have reported cardioprotective effects, suggesting that NO_3_^−^ sourced from vegetables confer benefits that outweigh traditional perceived risks [12,13]. Furthermore, to our knowledge, the highest dose of NO_3_^−^ investigated thus far is a dose of ~19.5 mmol of NO_3_^−^, which was tolerated by elite athletes [14].

**Figure 1:**
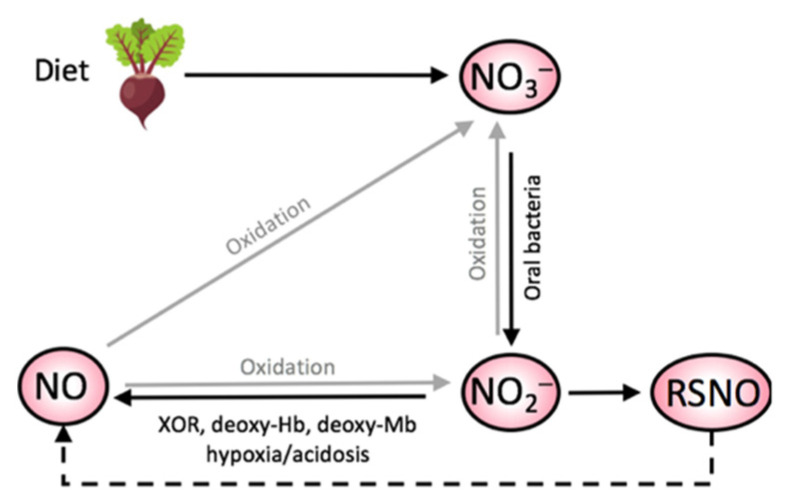
A schematic diagram of the nitrate–nitrite–nitric oxide (NO_3_^−^–NO_2_^−^–NO) pathway, which is facilitated by environments of low oxygen tension and acidosis. The conversion of NO_2_^−^ to NO occurs via various enzymatic reactions; S-nitrosothiols (RSNO) can decompose to form NO. XOR = xanthine oxidoreductase; deoxy-Hb = deoxyhemoglobin; deoxy-Mb = deoxymyoglobin.

The effects of dietary NO_3_^−^ ingestion are attributed to the elevation in nitric oxide (NO), a signaling molecule that regulates vascular, metabolic, and contractile processes [15], through the absorption and metabolism of NO_3_^−^ in the enterosalivary pathway [16]. Following consumption, exogenous NO_3_^−^ is absorbed by the gastrointestinal tract and enters the systemic circulation [16]. Upon reaching the salivary glands, NO_3_^−^ re-enters the oral cavity via protein transporters (e.g., sialin) and is concentrated in the saliva [16]. Consequently, anaerobic facultative bacteria that reside in the dorsal part of the tongue utilize salivary NO_3_^−^ for bacterial respiration [17] and reduce ~20% of NO_3_^−^ into nitrite (NO_2_^−^) (Figure 1). A portion of swallowed NO_2_^−^ is reduced into nitric oxide (NO) in the stomach [18], but the remaining NO_2_^−^ is transported to organs and tissues for storage or conversion into NO and S-nitrosothiols [19] via various NO_2_^−^ reducing proteins such as deoxyhemoglobin, deoxymyoglobin, and xanthine oxidoreductase [15]. Thus, it is commonly accepted that increased NO following dietary NO_3_^−^ ingestion is evidenced by a rise in plasma [NO_2_^−^] ([] denote concentration), the precursor molecule to NO [11]. Interestingly, this NO_3_^−^–NO_2_^−^–NO pathway prioritizes yielding NO in areas undergoing acidosis [20] or hypoxia [21] and, therefore, the areas that stand to benefit the most from NO. For example, contracting skeletal muscle during exercise exhibits these environments, providing a rationale to implement NO_3_^−^ supplementation strategies during exercise [22,23,24,25,26,27].

Dietary NO_3_^−^ has various proposed physiological mechanisms that may exert ergogenic effects, which include improved contractile function [28,29] c.f. [30], improved energy cost of force production [31], better homogenous blood flow distribution [32], and more pronounced effects in type II muscle fibers [28,32,33,34]. However, specific to power production, NO_3_^−^ supplementation may improve skeletal muscle force production and function by modulating calcium release [28,29] and/or sensitivity [35], particularly favoring fatigue-sensitive type II muscle fibers [28], although it should be noted that alterations to calcium-handling proteins have not been observed in humans [29,30]. The exact mechanism by which NO_3_^−^ may evoke structural changes to proteins regulating excitation-contraction coupling remains elusive but could be due to nitrosylation or molecular signaling via cyclic guanosine monophosphate [35]. Taken together, the mechanistic bases of dietary NO_3_^−^ supplementation support its efficacy in various exercise modalities and may suggest that exercise relying on high-velocity or high-power contractions, and thus a greater relative proportion of type II muscle fibers, would be improved following NO_3_^−^ supplementation. 

The current body of literature has evidenced that NO_3_^−^ can cause exercise performance enhancements in intermittent high-intensity exercise bouts [23,26], endurance exercise [27], and in some forms of resistance exercise [24], although it is acknowledged that discrepancies between studies exist [26,36]. For example, power output has been shown to improve with NO_3_^−^ in isokinetic dynamometry [37,38,39,40], as well as in cycling [41,42,43,44] and time to reach peak power [41,42,43,45]. More recently, a single dose of NO_3_^−^ improved the mean power (P_mean_) and mean velocity (V_mean_) of bench press by ~19% and ~7%, respectively [46]; however, there was no effect of NO_3_^−^ ingestion on bench press in another study [47]. Interestingly, performance has been reported to improve following NO_3_^−^ ingestion during exercise protocols likely recruiting a greater proportion of type II fibers [48], such as cycling at faster pedaling rates compared to slower pedaling rates [33,34] and isokinetic knee extensions at high angular velocities [37], reinforcing the possibility that NO_3_^−^ favors impacting high-velocity contractions. Furthermore, NO_3_^−^-induced improvements may predominate during the initial phases of contraction, as evidenced when power output was improved during shorter (6 s) maximal sprints compared to longer (30 s) maximal sprints [49]. In support of this notion, a recent meta-analysis by Wong et al. [36] interpreted that the peak power output (P_peak_) of single maximum sprinting efforts was more likely to be improved, but not during repeated maximum sprints, suggesting that the benefits of dietary NO_3_^−^ may be conferred better to contractions at earlier phases of force production. Together, the evidence supports the potential for dietary NO_3_^−^ in impacting powerful contractions with high velocities during short explosive efforts, but it is currently unclear how NO_3_^−^ may influence these movements, such as explosive efforts, emphasizing that further research is required. 

The aim of this review was to perform a summary of investigations that have investigated the effect of NO_3_^−^ supplementation in exercise involving explosive efforts (≤6 s) and to explore the factors that might have a role in the efficacy of NO_3_^−^ for explosive movements during various exercise modalities. 

## 2. Materials and Methods

### 2.1. Literature Search

The present review conducted a systematic search using the Preferred Reporting Items for Systematic Reviews and Meta-Analysis (PRISMA) guidelines [50] and was conducted for studies that investigated NO_3_^−^ supplementation on explosive exercise performance using PubMed, Scopus, and Directory of Open Access Journals (DOAJ) with no filter based on date of publication. The following terms were used: (nitrate OR nitrite OR beetroot) (concept 1) AND (supplement OR supplementation) (concept 2) AND (explosive OR power OR high intensity OR high-intensity OR sprint* OR “athletic performance”) (concept 3). A total of 18 articles met the eligibility criteria according to the Population, Intervention, Comparison, Outcomes, and Study (PICOS) framework (Table 1) for this systematic review. Two researchers independently checked all the search results and performed the screening and eligibility of studies. After contrasting outcomes between these two researchers, results showing discrepancy were evaluated by a third researcher. All documents that were not related with NO_3_^−^ supplementation or were not associated with the established PICOS criteria were discarded. 

### 2.2. Quality Assessment

A quality assessment was performed by two authors (L.C., R.D.) using the PEDro scale [51]. Following previous works [52], a 12th item was added to the scale: “Did the study assess the effectiveness of the blinding to the nitrate supplementation?”, in an attempt to adapt to the assessment of methodological quality and the specific characteristics of the studies included in this systematic review. Questions were answered with “yes” if the criteria were satisfied or with “no” if the criteria were not satisfied. Since PEDro’s first item is not included in the final score, the maximal score possible for one study was 11. Based on final scores, studies were classified as showing excellent (10–11 points), good (7–9 points), fair (5–6 points), or poor (<5 points) methodological quality [53,54]. Each study’s quality assessment was independently performed by two authors (L.C. and R.D.). Thereafter, these two researchers discussed and resolved by consensus any discrepancy, taking into consideration the opinion of a third author if no consensus was reached (Á.L.-R.).

### 2.3. Data Extraction

Two authors (L.C. and R.D.) independently extracted data from each study using a predefined data sheet, and they further discussed and resolved by consensus any potential discrepancy afterwards. The following information was compiled from each study: cite, information related to the sample (sample size, sex, level of physical fitness), supplementation protocol, exercise protocol, and performance outcomes. When a significant effect of NO_3_^−^ supplementation was reported (*p* ≤ 0.05), outcomes were presented according to the increase observed after supplementation, compared to placebo condition (expressed as %), which was calculated using the next equation: (mean value after supplementation condition—mean value after placebo condition)/mean value after placebo condition × 100. In addition, Cohen’s *d* and 95% confidence intervals are included in the results. When values for Cohen’s *d* calculations were not reported in the original article, it was calculated based on the available data. When data were unavailable for calculation, Cohen’s *d* was reported as ‘unknown’. Effect sizes were defined as trivial (<0.2), small (<0.5), moderate (<0.8), and large (>0.8) [55].

## 3. Results

### 3.1. Study Selection

**Figure 2:**
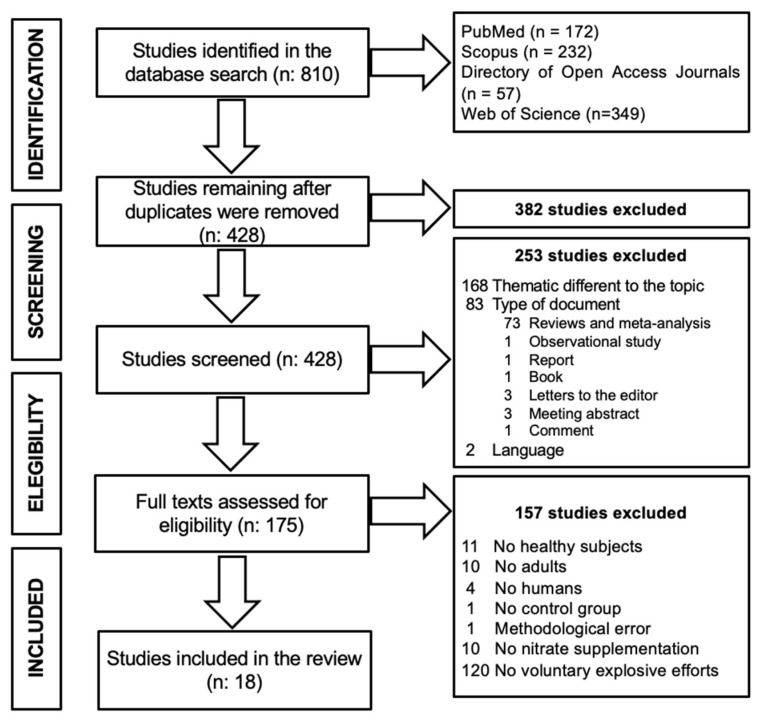
Preferred Reporting Items for Systematic Reviews and Meta-Analysis (PRISMA) flowchart [58].

A total of 810 results were initially obtained, which were reduced to 428 results after excluding duplicates (*n* = 382). After excluding articles that were not related to the topic or did not comply with the type of document of interest of the present study (*n* = 253), a total of 175 manuscripts were originally identified for this review. After screening for inclusion criteria, 157 articles were excluded since they did not evaluate healthy participants (*n* = 25), did not test a control group (*n* = 1), presented methodological errors (*n* = 1), did not use NO_3_^−^ supplementation (*n* = 10), and/or did not assess voluntary explosive efforts (*n* = 120). A total of 18 articles were finally selected for this systematic review (Figure 2). Of the 18 studies selected, 2 studies [56,57] had exercise protocols that included both sprint and resistance explosive efforts and, thus, were divided into both Table 2 and Table 3 for the corresponding type of exercise.

The experimental design and results of all reviewed studies in this article are provided in Table 2 and Table 3, while the quality of assessments is provided in Table 4. The exercise modalities used to assess explosive exercise performance included sprint- and/or resistance-based exercise efforts that were ≤ 6 s [2]. Recovery periods ranged between 16 s [59] and 2 min [44]. 

A total of 18 studies were selected for this systematic review (Figure 2), which evaluated a total sample of 267 participants. Only 20 females were tested, with the remaining 247 being males. Out of these 267 participants, 228 were moderately active [37,40,46,49,57,59,60,61,62,63,64,65,66,67], 13 were elite athletes [44], and 26 did not practice any sport [56,68]. A total of eight studies assessed the effects of NO_3_^−^ supplementation in explosive resistance efforts [37,40,46,47,65,66,67,68], whereas another eight evaluated explosive sprint-type efforts [44,49,59,60,61,62,63,64], with the remaining two studies performing a combination of both tests [56,57].

### 3.2. Study Characteristics

Sprint-based exercises were performed using cycle ergometers [44,49,59,62,63], running lanes [60], a tennis court [57], and a treadmill [56], with locations such as an indoor gymnasium [61] or in the laboratory [64]. Buck et al. [61] and Thompson et al. [59] performed a team sport simulation test. López-Samanes et al. [57] assessed the speed of tennis serves and evaluated an agility test. The remaining studies [56,57,60,64] employed sprints, without other types of exercise protocols. Among the sprints performed on a cycle ergometer, two were tested under specific environmental conditions, such as hypoxia [63] or elevated temperature and humidity [62]. 

Resistance-based exercise protocols included half squats using a flywheel device [65], bench press using a Smith machine [47], back squat and bench press using free weights [46], isometric [40,57,66,67,68] or isokinetic [37,40,56,67] knee flexions and extensions, isometric handgrip contractions [57], and countermovement jumps (CMJ) [40,57]. The recovery periods between efforts ranged from 15 s [68] to 3 min [46,65].

With the exception of Kramer et al. [67], in which potassium NO_3_^−^ salts (KNO_3_) were administered, beetroot juice was the source of NO_3_^−^ for all remaining studies. All placebo interventions used in the reviewed studies had negligible NO_3_^−^ content. In most cases [37,40,44,49,59,61,62,63,65,66] concentrated beetroot juice was administered for the experimental and placebo condition, where the placebo consisted of NO_3_^−^-depleted concentrated beetroot juice. In other studies, the placebo was administered as blackcurrant juice [46,47,56,68], potassium chloride tablets [67], beetroot juice diluted in water [57], and juices with a similar caloric and macronutrient content (but different in antioxidants, texture, and taste) in the control group [64]. 

Both acute [37,44,46,47,57,62,63,65] and chronic [40,49,59,60,61,64,66,67,68] supplementation protocols were employed in the reviewed studies. NO_3_^−^ dosing ranged from 6.2 mmol [62] to 12.9 mmol [62,65] of NO_3_^−^ for acute protocols, and from 6 mmol [61] to 15.8 mmol [40] for chronic protocols. NO_3_^−^ supplementation was administered 2–3 h before testing for the acute protocols, whereas multi-day NO_3_^−^ supplementation occurred in a range of short-term (3 days) [59,64] to chronic (7 days) [59,66,68] strategies before the onset of testing.

## 4. Discussion

This is the first systematic review to have examined whether dietary NO_3_^−^ supplementation can enhance exercise performance during explosive efforts lasting for ≤6 s in various modes of exercise. The main finding was that dietary NO_3_^−^ ingestion has the potential to increase power output, sprint time, and total work in sprint-type exercise, as well as power and velocity of contraction in resistance-type exercise, but that these effects are likely impacted by factors affecting NO bioavailability, exercise modality, and participant characteristics.

### 4.1. The Effects of Dietary Nitrate on Explosive Sprinting Exercise Performance

Out of the 10 studies that investigated explosive efforts during sprinting exercise, there were 5 studies that administered short-term (3 days) and chronic (≥3 days) dietary NO_3_^−^ supplementation [49,59,60,61,64], while the other 5 studies provided acute NO_3_^−^ ingestion (~2–3 h prior to exercise) [44,56,57,62,63]. There were four studies that observed significant performance improvements to sprints in cycling [44,49,59] and running [60]. Specifically, NO_3_^−^ improved cycling maximal power (P_max_) [44], optimal repetitions per minute (RPM_opt_) [44], P_mean_ [49], and total work performed [59]. Interestingly, three of these studies provided NO_3_^−^ chronically, ranging from 3 [49,60] to 7 days [59], whilst Rimer et al. [44] administered NO_3_^−^ acutely ~2–3 h prior to exercise. However, there were two studies that administered chronic NO_3_^−^ supplementation that did not report improvements to sprint times [61,64]. Clifford et al. [64] provided 3 days of NO_3_^−^ supplementation to healthy males, but importantly, the NO_3_^−^ dose reported (143 mg or ~2.3 mmol of NO_3_^−^) is markedly lower than the findings of a dose–response study, where 8.4 mmol of NO_3_^−^ was evidenced to be the minimum amount required to induce physiological effects [11]. 

There is evidence that the magnitude of elevation in plasma [NO_2_^−^] (and thus NO bioavailability) is associated with improvements to muscular work and capacity [25,39,69,70], but it is acknowledged that other factors may have a role in a lack of effect. In support of this notion, with 6 days of NO_3_^−^ supplementation, Buck et al. [61] did not observe improvements to sprint time in female amateur team sport players, but it could be possible that the experiment was underpowered given that the power calculation was based on an experiment with a rowing exercise protocol [71] when the study employed running sprints. For example, running sprints were improved in Thompson et al. [60] where 36 participants were recruited compared to the 13 participants recruited in Buck et al. [61]. Furthermore, it could be possible that the inclusion of female participants (wherein nine females were not on hormonal contraceptives) had a role in the discrepancy. Importantly, chronic NO_3_^−^ ingestion is likely not the sole factor in determining the efficacy of NO_3_^−^ in performance during explosive sprinting exercise. However, given that most studies that did not observe effects employed acute NO_3_^−^ dosing regimens, these data could highlight supplementation duration as a methodological consideration for NO_3_^−^-induced effects on explosive sprinting performance. Together, these observations could suggest that explosive sprint efforts in cycling and/or running could require consecutive multi-day NO_3_^−^ loading to induce performance effects. Clearly, further research is warranted to understand the optimal dosing guidelines for NO_3_^−^ supplementation and whether there is an influence on explosive sprinting efforts.

### 4.2. The Effects of Dietary Nitrate on Explosive Resistance Exercise Performance

Out of the 10 studies that employed explosive efforts during resistance-type exercise, 6 of the studies administered NO_3_^−^ acutely (2 to 3 h prior to exercise) while 4 of the studies administered chronic NO_3_^−^ supplementation over 6 days [40] or 7 days [66,68]. There were four studies that reported performance improvements to peak power (P_peak_) in squat exercise using a fly wheel device (+12–22%) [65] isokinetic knee flexion at 60°/s (+2%) [40], isokinetic knee extension at 6.28 rad/s (+4%) [37], and during free-weight bench press (+19%) [46]. In addition, the mean velocity (V_mean_) of contraction was enhanced (+7%) during free-weight bench press exercise [46]. Interestingly, three of the four studies that observed performance enhancements in resistance-type exercise employed acute NO_3_^−^ supplementation [38,46,65]. These data are in contrast to the supplementation regimens (short-term and chronic NO_3_^−^ supplementation) reported in studies that observed improved explosive efforts during sprinting exercise following NO_3_^−^ supplementation. Moreover, despite two of the studies providing the same NO_3_^−^ dose (1 × 70 mL of 6.4 mmol of NO_3_^−^) for bench press exercise, Ranchal-Sanchez et al. [47] did not observe any improvements to power or velocity of contraction at 60%1RM, 70%1RM, or 80%1RM, compared to the ~19% and ~7% improvement to power and velocity of bench press at 70%1RM [46] following NO_3_^−^ ingestion. In knee extension and flexion exercise protocols, there is conflicting data given that acute [56] and chronic NO_3_^−^ supplementation (6–7 days) did not impact peak torque (T_peak_) [67] or maximal force (F_max_) [66,68], but flexion [40] and extension [37] at specific angular velocities exhibited a ~2–4% improvement to P_peak_. Lastly, handgrip strength [57] and countermovement jump (CMJ) performance [40,57] were not influenced by NO_3_^−^. These discrepancies are difficult to explain given the limited evidence available surrounding the effects of NO_3_^−^ ingestion on resistance exercise, and there are considerable differences in study designs between the reviewed studies. However, potential factors influencing the efficacy NO_3_^−^ on sprint- and resistance-type explosive exercise are discussed below.

### 4.3. Potential Factors Influencing Nitrate-Induced Improvements in Explosive Exercise

It is unclear as to why improved explosive efforts in sprint-based exercise occurred in more studies with short-term and chronic NO_3_^−^ supplementation dosing regimens, whereas improved explosive efforts in resistance-based exercise occurred in more studies with acute NO_3_^−^ dosing strategies. However, these data highlight that exercise modality is likely a factor that influences the efficacy of dietary NO_3_^−^ supplementation and emphasizes the need for further research in this area. In the studies that incorporated weightlifting, the participants were resistance-trained compared to the team sport players of the studies with sprinting exercise; thus, it could be possible that differences in the training history of participants impacted motor unit recruitment patterns [48] and muscle fiber type composition [72,73]. This is in line with recent speculation that NO_3_^−^ supplementation has a greater efficacy during the initial phases of contraction during force production [36,41,42,43] and thus the speed of acceleration of movements, which could be training-dependent. Other factors such as biomechanics, motor skills, delivery of instructions, and equipment might contribute to influence the efficacy of NO_3_^−^ on performance between explosive sprint and resistance exercise [74]. Furthermore, differences in the recruitment of upper and lower body musculature during exercise [71] may be a factor to explore given that fiber type seems to impact the efficacy of NO_3_^−^ on physiology [28,29].

#### 4.3.1. Nitric Oxide Bioavailability: Supplementation Strategies

The efficacy of dietary NO_3_^−^ supplementation is thought to be linked to the magnitude of elevation in NO bioavailability, which could partly account for the discrepancies in the literature and reviewed studies above. NO bioavailability is often measured through the surrogate marker of plasma [NO_2_^−^], such that a greater increase in plasma [NO_2_^−^] has been associated with improved muscular work or capacity [39,69,70]. However, there are several possible factors that influence NO bioavailability such as dosing regimen (i.e., timing, acute vs. chronic and total NO_3_^−^ provided), where it is now widely accepted that a dose of >6 mmol of NO_3_^−^ is required [11] and that there may be additional performance benefits with larger doses [25,69]. In addition, the vehicle of administration (i.e., juices vs. salts) could be influential on the efficacy of NO_3_^−^ given that NO_3_^−^-rich beetroot juice contains bioactive phytochemicals such as flavonoids, betalains, and ascorbic acid [75]. Although speculative, the presence of these antioxidant constituents could further elevate NO given that polyphenols facilitate the reduction of NO_2_^−^ to NO [76] or induce synergistic effects [15]. Flueck et al. [77] reported that the administration of NO_3_^−^-rich beetroot juice improved oxygen consumption during exercise compared to NO_3_^−^ salts, supporting the notion that juices may be more effective, but further work is required. The training status of participants [78] may be an influential factor given that redox status impacts NO bioavailability [79] and that baseline plasma [NO_2_^−^] could be higher in elite athletes due to differences in habitual diet in highly trained populations [80]. Lastly, it is unknown whether NO bioavailability (or other physiological mechanisms) is influenced when dietary NO_3_^−^ is co-ingested with other ergogenic aids. In the limited available data thus far, no synergistic effects on exercise performance have been reported when beetroot juice was co-ingested with caffeine [81,82,83] or sodium bicarbonate [84], but further research is warranted to understand whether combining multiple ergogenic aids or other nutritional strategies with dietary NO_3_^−^ could impact the effects of dietary NO_3_^−^ on performance in various exercise modalities and participant populations.

#### 4.3.2. Nitric Oxide Bioavailability: Skeletal Muscle Modulations and Storage

The effects of multi-day NO_3_^−^ ingestion may be important for NO bioavailability by providing a time window for structural and functional changes to proteins responsible for excitation–contraction coupling [35]. For example, acute NO_3_^−^ ingestion could induce signaling effects to alter myofibrillar protein function [15], but NO_3_^−^ loading over several days could potentially provide further augmentations through increased NO_3_^−^ content stored in skeletal muscle [85]. In addition, multi-day NO_3_^−^ loading could alter expression and/or function of proteins regulating calcium release from the sarcoplasmic reticulum [28,29]. However, to date, chronic NO_3_^−^ supplementation has not been observed to change calcium handing proteins in humans [30], and most data on skeletal muscle NO_3_^−^ and NO_2_^−^ are from rodent studies [86,87,88]. The ability for skeletal muscle to serve as a NO_3_^−^ storage site is an attractive notion given that the NO_3_^−^–NO_2_^−^–NO pathway would enable manipulation of stored muscle NO_3_^−^ through the ingestion of dietary NO_3_^−^ and, thus, potentially improve local utilization of NO from NO_3_^−^ reserves. In humans, recent advances show that NO_3_^−^ content is significantly greater in skeletal muscle compared to plasma, and that ingestion of ~13 mmol of NO_3_^−^ increased muscle NO_3_^−^ by five-fold [89]. Moreover, the skeletal muscle NO_3_^−^ concentration declined by 39% following high-intensity exercise to exhaustion, highlighting the potential role of muscle in localized NO production during exercise [89]. Further work is required to understand the mechanistic bases of NO_3_^−^ in muscle function, as well as the role of skeletal muscle NO_3_^−^ and whether altering muscle NO_3_^−^ content is associated with improvements to muscle function and exercise performance.

#### 4.3.3. Nitric Oxide Bioavailability: Oral Microbiome

The role of the oral microbiome in enhancing NO bioavailability is not well understood but may have important implications for the NO_3_^−^–NO_2_^−^–NO pathway and thus NO homeostasis [90]. For example, the use of oral hygiene products (e.g., mouthwash [91,92]) or elevation in thiocyanate, a compound competing NO_3_^−^ transporters (e.g., smoking [93]), has been evidenced to disrupt NO_3_^−^ metabolism, consequently attenuating the characteristic elevation in plasma [NO_2_^−^] following NO_3_^−^ ingestion and abolishing potential positive NO_3_^−^-induced effects, such as the lowering of blood pressure [93]. Recent advances suggest that NO_3_^−^ ingestion could favorably alter the microbial composition such that NO_3_^−^ reducing taxa are increased [94], and thus it could be reasoned that, consequently, elevations in NO_2_^−^ could be more pronounced following multi-day ingestion of NO_3_^−^ as microbial composition is modulated. However, the exact impact of NO_3_^−^ dose and duration, as well as the oral microbiota species on influencing NO bioavailability and muscle physiology, is still elusive and requires further research.

#### 4.3.4. Nitric Oxide Bioavailability: Sex Differences

In the reviewed studies, 20 out of 267 participants were female, which is equivalent to only ~7% of the total number of participants as females. It is extremely unfortunate that females have been underappreciated in sports and exercise medicine research for so long [95], and thus it is unsurprising that the impact of sex differences on the efficacy of dietary NO_3_^−^ remains unexplored. However, the scientific community is progressively becoming cognizant of potential sex differences in NO_3_^−^ research, and open questions for investigation include examining sex-based physiological [96] and hormonal [97] influences on the mechanisms and performance effects of NO_3_^−^. To date, only eight studies have exclusively included females in dietary NO_3_^−^ research, and there are inconsistencies between studies regarding the control for the menstrual cycle and hormonal contraceptives [see review: 96]. These controls are further complicated as there is currently no consensus on whether oral contraceptive use and/or the menstrual cycle affect strength and power performance, thus potentially under-powering studies that assess explosive power in female-only cohorts. One important sex difference was reported by Kapil et al. [98] such that the oral microbiome in females has an increased capacity to reduce NO_3_^−^ into NO_2_^−^. However, whether this physiological difference has an influence on the bioavailability and bioactivity of NO and its subsequent effects on NO_3_^−^-induced mechanisms and performance have yet to be elucidated, making this a ripe area for research. Lastly, although hormonal controls may be barriers to include females, scientists are encouraged to examine if their laboratories are capable of incorporating as many methodological considerations for testing females if possible [97].

#### 4.3.5. Methodology

In the selection of an exercise protocol during the study design phase, the tests have to be feasible for acquiring relevant physiological mechanistic data and to sufficiently induce the correct metabolic demand for the sport discipline of interest. It is important to note that the reliability and validity of exercise tests (test–retest repeatability over multiple trials) is crucial for the detection of ‘real’ changes between an intervention and control group, especially for small performance changes, as well as to minimize inaccurate interpretations of results. However, there is a paucity of repeatability data reported within dietary NO_3_^−^-research [59] and in the literature surrounding various high-intensity protocols [99,100,101,102,103]. Furthermore, given the obvious translation of high-intensity exercise protocols to numerous sports, there are infinite work-to-rest combinations for designing high-intensity exercise protocols, requiring many validation studies to account for each protocol. Thus far, the repeatability of data may be improved by the inclusion of familiarization trials [99], as some of the reviewed studies employed [44,49,59,62,63]. In other studies [61,62], authors employed exercise protocols based on previously validated tests [104,105], although few provide citations. Future studies may consider including repeatability data to provide better insight on how translatable the results are. Moreover, in publications without repeatability data (i.e., intra- and inter-individual variability), it is important to be cognizant of extrapolating the results beyond reasonable conclusions.

### 4.4. Candidate Physiological Mechanisms

To date, several physiological mechanisms have been reported to underpin NO_3_^−^-induced performance enhancements, and the relative contribution of each mechanism may depend on the circumstance, participant population, and exercise protocol. Skeletal muscle function has been shown to increase in force production following 7 days of NO_3_^−^ supplementation in rodents, in which the authors attributed to increased protein expression in type II muscle fibers of dihydropyridine receptors and calsequestrin, as well as increased intracellular calcium concentration [28]. More recently, exercise tolerance was improved in rodents due to preserved calcium handling protein function [29]. However, in humans, Whitfield et al. [30] did not observe changes to any calcium handling protein content following 7 days of NO_3_^−^ supplementation, which is in contrast to the rodent data [28,29]. However, since cysteine residues exist on many of the proteins that regulate excitation–contraction coupling [106], it could be possible that these proteins undergo the process of S-nitrosylation [19] in altering function during contraction without requiring any change in protein expression. Moreover, NO_3_^−^ supplementation has been observed to modulate hydrogen peroxide [107], a reactive oxygen species molecule that has potential to alter force production independent from calcium via inducing signaling cascades [79]. Taken together, it remains a possibility that dietary NO_3_^−^ supplementation evokes physiological effects and performance enhancements by modulating the regulatory processes of calcium release and reuptake and/or by altering skeletal muscle redox status. 

Dietary NO_3_^−^ supplementation has also been evidenced to influence the phosphocreatine system such that the phosphocreatine cost of energy production was lower and accompanied by an attenuated accumulation of metabolites during knee extensor exercise [31]. Although the exact mechanisms are unclear, by preserving phosphocreatine, it is possible that NO_3_^−^ impacts the efficiency of this system and thus would benefit exercise protocols relying on the phosphocreatine system, such as explosive contractions. Furthermore, the blood flow distribution has been observed to improve between muscle fiber types following NO_3_^−^ supplementation [32], which could suggest that recovery between high-intensity explosive repetitions may be improved and thus could preserve power across several repetitions. However, it is notable that NO_3_^−^ supplementation has not been observed to impact phosphocreatine resynthesis unless in hypoxic conditions [108]. 

## 5. Conclusions

In conclusion, dietary NO_3_^−^ supplementation may be effective in improving the power and velocity of explosive resistance exercise efforts such as weightlifting, as well as the total work, and sprint time of explosive repeated sprint-type exercise protocols in cycling and running. However, these effects may be dependent on the NO_3_^−^ supplementation regimen, participant characteristics, and exercise modality. Furthermore, to improve the interpretation of results and to minimize false conclusions, future studies are encouraged to report the validity and reliability of the exercise protocols employed, given that relatively small differences may be observed. Clearly, more research is required to elucidate the impacts of manipulating dosing regimens in various exercise modalities and in females to understand how and when to administer NO_3_^−^ supplementation for optimizing NO bioavailability to enhance explosive movements.

## Figures and Tables

**Table 1:** Inclusion criteria according to the Population, Intervention, Comparison, Outcomes, and Study (PICOS) framework.

Parameter	Inclusion Criteria
Population	Adult healthy population
Intervention	Acute and/or chronic Supplementation with NO_3_^−^
Comparison	A placebo condition (supplementation depleted on NO_3_^−^)
Outcome	Variables related to performance of explosive efforts (≤6 s [2])
Setting	Randomized double-blind placebo-controlled studies

**Table 2:** Description of studies that have investigated the effects of dietary NO_3_^−^ supplementation on high-intensity, sprint-type explosive efforts in humans. There were 4 studies out of 10 that observed significant performance improvements to sprints in cycling [44,49,59] and running [60]. Specifically, NO_3_^−^ improved cycling P_max_ [44], RPM_opt_ [44], P_mean_ [49], and total work performed [59].

Reference	Subjects	Supplementation	Exercise Protocol	Results
Buck et al. [61]	13 female amateur basketball and soccer players	6 d of BR supplementation (NO_3_^−^ 6 mmol per day)	Before, during, and after 60 min in a simulated team-game: 6 × 20 m running sprints, 25 s rest	↔ Best sprint time (set 1): −0.3% (3.68 ± 0.26 vs. 3.69 ± 0.25 s; d = 0.04 [−0.85 to 0.77]) ↔ Best sprint time (set 2): −0.8% (3.77 ± 0.32 vs. 3.80 ± 0.25 s; d = 0.11 [−0.91 to 0.71]) ↔ Best sprint time (set 3): +1.1% (3.81 ± 0.32 vs. 3.77 ± 0.25 s; d = 0.14 [−0.68 to 0.95])
Rimer et al. [44]	13 competitive athletes (female, *n* = 2, male, *n* = 11)	2.5 h prior to exercise acute BR ingestion (NO_3_^−^ 11.2 mmol)	4 × 3 to 4 s cycling sprints, 2 min rest	↑ Pmax: +6 ± 2.6 vs. 2 ± 3.8% (d = 1.21 [0.31 to 2.07]) ↑ RPMopt: +6.5 ± 11.4 vs. 0.3 ± 4.1% (d = 0.79 [−0.14 to 1.54])
Smith et al. [62]	12 male recreationally active athletes	3 h prior to exercise acute BR ingestion (NO_3_^−^ 6.2 mmol)	20 × 6 s cycling sprints in temperate (22.5 °C) and hot environmental conditions (30 °C), 114 s rest	↔ Ppeak (hot): −6.0% (659 ± 100 vs. 683 ± 139 W; d = 0.21 [−1.04 to 0.66]) ↔ Pmean (temperate): −1.6% (562 ± 120 vs. 571 ± 124 W; d = 0.08 [−0.92 to 0.78]) ↔ Pmean (hot): −5.9% (543 ± 29 vs. 575 ±39 W; d = 0.97 [−11.79 to −0.01]) ↔ Total work (temperate): −1.5% (67.44 ± 14.39 vs. 68.46 ± 15.07 kJ; d = 0.07 [−0.91 to 0.78]) ↔ Total work (hot): −5.6% (66.07 ± 10.84 vs. 69.74 ± 15.13 kJ; d = 0.03 [−1.12 to 0.58])
Thompson et al. [59]	16 male recreational team-sport players	2.5 h prior to exercise and 7 d of BR supplementation (NO_3_^−^ 12.8 mmol per day)	2 × 40 min cycling sprints, 15 min rest 10 × 6 s sprints, 100 s rest at 35% VO_2max_ + 14 s passive rest 5 × 4 s sprints, 16 s rest at 35% VO_2max_ 10 × 6 s sprints, 100 s rest at 35% VO_2max_ + 14 s passive rest	↑ Total work: +3.5% (123 ± 19 vs. 119 ± 17 kJ; d = 0.23 [−0.51 to 0.94])
Wylie et al. [49]	10 male recreational team-sport players	2.5 h prior to exercise and 3 to 5 d of BR supplementation (NO_3_^−^ 8.2 mmol per day)	24 × 6 s cycling sprints, 24 s rest	↔ Ppeak (mean): +1.3% (792 ± 159 vs. 782 ± 154 W; d = 0.07 [−0.88 to 1.00]) ↑ Pmean (sprints 1–6): +7.3% (694 ± 125 vs. 647 ± 122 W; d = 0.39 [−0.58 to 1.31]) ↔ Pmean (sprints 7–12): +3.9% (560 ± 100 vs. 539 ± 112 W; d = 0.20 [−0.75 to 1.13]) ↔ Pmean (sprints 13–18): +5.3% (518 ± 111 vs. 492 ± 121 W; d = 0.23 [−0.73 to 1.16]) ↔ Pmean(sprints 19–24): +4.8% (500 ± 114 vs. 477 ± 119 W; d = 0.20 [−0.75 to 1.13]) ↑ Pmean (mean): +5.4% (568 ± 136 vs. 539 ± 136 W; d = 0.22 [−0.74 to 1.15])
Kent et al. [63]	12 male team sport players	2 h prior to exercise acute BR ingestion (NO_3_^−^ 13 mmol)	4 sets of 9 × 4 s cycling sprints with 16 s active + 6 s passive rest, interspersed with 3 min rest (3000 m simulated altitude)	↔ P_peak_ (set 1): −2.4% (1185 ± 172 vs. 1214 ± 179 W; d = 0.17 [−1.01 to 0.69]) ↔ P_peak_ (set 2): −1.0% (1157 ± 178 vs. 1181 ± 163 W; d = 0.15 [−0.98 to 0.71]) ↔ P_peak_ (set 3): −0.5% (1159 ± 186 vs. 1165 ± 160 W; d = 0.04 [−0.88 to 0.81]) ↔ P_peak_ (set 4): −1.0% (1152 ± 194 vs. 1164 ± 139 W; d = 0.07 [−0.92 to 0.78]) ↔ P_mean_ (set 1): −2.7% (807 ± 144 vs. 829 ± 144 W; d = 0.16 [−1.00 to 0.70]) ↔ P_mean_ (set 2): +3.1% (794 ± 156 vs. 770 ± 142 W; d = 0.17 [−0.69 to 1.00]) ↔ P_mean_ (set 3): +2.1% (809 ± 150 vs. 792 ± 131 W; d = 0.15 [−0.73 to 0.96]) ↔ P_mean_ (set 4): −1.0% (779 ± 156 vs. 804 ± 122 W; d = 0.19 [−1.02 to 0.68]) ↔ Total work (set 1): −2.7% (29.0 ± 5.18 vs. 29.8 ± 5.19 J; d = 0.16 [−1.00 to 0.70]) ↔ Total work (set 2): −0.5% (28.5 ± 5.61 vs. 28.7 ± 5.10 J; d = 0.04 [−0.88 to 0.81]) ↔ Total work (set 3): +2.5% (29.1 ± 5.38 vs. 28.4 ± 4.75 J; d = 0.14 [−0.71 to 0.98]) ↔ Total work (set 4): +0.2% (28.9 ± 5.62 vs. 28.9 ± 4.39 J; d = 0.00 [−0.85 to 0.85]) ↔ Work decrement (set 1): −47.1% (11.9 ± 6.9 vs. 17.5 ± 11.7%; d = 0.48 [−1.43 to 0.30]) ↔ Work decrement (set 2): −21.7% (12.9 ± 9.1 vs. 15.7 ± 14.5%; d = 0.47 [−1.07 to 0.63]) ↔ Work decrement (set 3): +4.5% (13.9 ± 8.4 vs. 13.3 ± 11%; d = 0.09 [−0.79 to 0.91]) ↔ Work decrement (set 4): +2.5% (12.2 ± 6.3 vs. 11.9 ± 7.1%; d = 0.05 [−0.80 to 0.89])
Kokkinoplitis et al. [56]	7 healthy males	3 h prior to exercise acute BR ingestion (NO_3_^−^ 6.45 mmol)	5 × 6 s running sprints on treadmill, 30 s rest	↔ P_peak (mean)_: +4.9% (4133.5 ± 674.4 vs. 3938.3 ± 603.1 W; d = 0.33 [−0.89 to 1.46])
Thompson et al. [60]	36 team sport players	2.5 h prior to exercise and 5 d of BR supplementation (12.8 mmol NO_3_^−^ per day)	5 × 20 m running sprints in running lanes, 30 s rest	↓ Total time: −1.2% (3.98 ± 0.18 vs. 4.03 ± 0.19 s; d = 0.27 [−0.71 to 0.20]) ↓ Time (5 m): −2.3% (1.73 ± 0.09 vs. 1.77 ± 0.09 s; d = 0.45 [−0.92 to 0.04]) ↓ Time (10 m): −1.6% (2.53 ± 0.2 vs. 2.57 ± 0.12 s; d = 0.25 [−0.71 to 0.23]) ↓ Time (5–10 m): −1.2% (0.80 ± 0.04 vs. 0.81 ± 0.04 s; d = 0.25 [−0.72 to 0.22]) ↔ Time (10–20 m): −0.7% (1.45 ± 0.07 vs. 1.46 ± 0.09 s; d = 0.13 [−0.59 to 0.35])
Clifford et al. [64]	20 male team sport players	4 d of BR supplementation (2.31 mmol NO_3_^−^ per day)	20 × 30 m sprints, 30 s rest	↔ Best sprint time: BR −0.7% (4.38 ± 0.17 vs. 4.41 ± 0.23 s; d = 0.15 [−0.79 to 0.50]) and PL +1.1% (4.53 ± 0.15 vs. 4.48 ± 0.14 s; d = 0.35 [−0.31 to 0.98]).
López-Samanes et al. [57]	13 trained male tennis players	3 h prior to exercise of acute BR ingestion (6.4 mmol NO_3_^−^)	5 tennis serves, 2 × 10 m sprints, 2 × agility test (5–0–5), 1 min rest	↔ Serve speed: −2.7% (160.6 ± 10.4 vs. 165.0 ± 10.8 km/h; d = 0.15 [−1.22 to 0.42]) ↔ Best sprint time (10 m): +1.1% (1.86 ± 0.07 vs. 1.88 ± 0.05 s; d = 0.39 [−1.13 to 0.50]) ↔ Best sprint time (5–0–5): +2.0% (2.60 ± 0.10 vs. 2.64 ± 0.10 s; d = 0.69 [−1.21 to 0.43])

↑ = significant increase; ↓ = significant decrease; ↔ = no difference; BR = beetroot juice; *n* =sample size; NO_3_^−^ = nitrate; PL = placebo; P_max_ = maximal power; P_mean_ = mean power; P_peak_ = peak power; RPM_opt_ = maximal optimal pedaling rate; VO_2max_ = maximal oxygen consumption; [ ] denotes confidence intervals.

**Table 3:** Description of studies that have investigated the effects of NO_3_^−^ supplementation on explosive resistance efforts in humans. There were 4 studies out of 10 that reported improved P_peak_ in back squat [65], isokinetic knee flexion at 60°/s [40], isokinetic knee extension at 6.28 rad/s [37], and during free-weight bench press [46] as well as V_mean_ of contraction during free-weight bench press exercise [46].

Reference	Subjects	Supplementation	Exercise Protocol	Results
Ranchal-Sánchez et al. [47]	12 resistance-trained male athletes	2 h prior to exercise acute BR ingestion (NO_3_^−^ 6.4 mmol)	2 × concentric Smith-machine back squats and bench press at 60%, 70%, and 80% 1RM, 2 min rest	↔ Pmax (60% 1RM) squat: +1.8% (389 ± 117 vs. 382 ± 111 W; d = 0.06 [−0.79 to 0.91]) ↔ Pmax (70% 1RM) squat: −0.5% (393 ± 116 vs. 395 ± 107 W; d = 0.02 [−0.83 to 0.83]) ↔ Pmax (80% 1RM) squat: −0.3% (377 ± 108 vs. 378 ± 96 W; d = 0.01 [−0.86 to 0.84]) ↔ Pmax (60% 1RM) bench press: −1.0% (289 ± 88 vs. 292 ± 94 W; d = 0.03 [−0.88 to 0.81]) ↔ Pmax (70% 1RM) bench press: +1.7% (242 ± 81 vs. 238 ± 81 W; d = 0.05 [−0.80 to 0.89]) ↔ Pmax (80% 1RM) bench press: −8.5% (176 ± 66 vs. 191 ± 55 W; d = 0.26 [−1.09 to 0.61]) ↔ Vmax (60% 1RM) squat: +1.8% (0.70 ± 0.09 vs. 0.69 ± 0.09 m/s; d = 0.14 [−0.74 to 0.95]) ↔ Vmax (70% 1RM) squat: +0.0% (0.61 ± 0.08 vs. 0.61 ± 0.08 m/s; d = 0.08 [−0.85 to 0.85]) ↔ Vmax (80% 1RM) squat: +0.0% (0.51 ± 0.09 vs. 0.51 ± 0.06 m/s; d = 0.01 [−0.85 to 0.85]) ↔ Vmax (60% 1RM) bench press: +0.0% (0.61 ± 0.08 vs. 0.61 ± 0.08 m/s; d = 0.04 [−0.85 to 0.85]) ↔ Vmax (70% 1RM) bench press: +0.0% (0.43 ± 0.06 vs. 0.43± 0.08 m/s; d = 0.03 [−0.85 to 0.85]) ↔ Vmax (80% 1RM) bench press: −9.7% (0.28 ± 0.05 vs. 0.31 ± 0.05 m/s; d = 0.62 [−1.45 to 0.29])
Rodríguez-Fernández et al. [65]	18 trained male athletes	2.5 h prior to exercise acute BR ingestion (NO_3_^−^ 12.9 mmol)	4 × 8 half squat in a flywheel device (0.025, 0.05 and 0.100 kg/m^2^) with 3 min of rest	↑ P_peak CON_ (0.025 kg/m^2^): +16.4% (1251 ± 249 vs. 1075 ± 205 W; d = 0.79 [0.05 to 1.46]) ↑ P_peak ECC_ (0.025 kg/m^2^): +18.9% (1195 ± 265 vs. 1005 ± 176 W; d = 0.87 [0.12 to 1.53]) ↑ P_peak CON_ (0.050 kg/m^2^): +15.3% (1182 ± 226 vs. 1025 ± 181 W; d = 0.79 [0.05 to 1.45]) ↑ P_peak ECC_ (0.050 kg/m^2^): +12.9% (1168 ± 261 vs. 1034 ± 172 W; d = 0.62 [−0.10 to 1.29]) ↑ P_peak CON_ (0.075 kg/m^2^): +20.8% (1132 ± 239 vs. 937 ± 158 W; d = 0.99 [0.23 to 1.66]) ↑ P_peak ECC_ (0.075 kg/m^2^): +19.7% (1201 ± 261 vs. 1003 ± 187 W; d = 0.90 [0.19 to 1.20]) ↑ P_peak CON_ (0.100 kg/m^2^): +18.4% (1008 ± 197 vs. 851 ± 161 W; d = 0.90 [0.14 to 1.56]) ↑ P_peak ECC_ (0.100 kg/m^2^): +12.0% (1070 ± 230 vs. 955 ± 191 W; d = 0.56 [−0.16 to 1.22]) ↑ P_mean CON_ (0.025 kg/m^2^)_:_ +16.4% (750 ± 173 vs. 644 ± 153 W; d = 0.67 [−0.06 to 1.33]) ↑ P_mean ECC_ (0.025 kg/m^2^)_:_ +19.6% (684 ± 154 vs. 572 ± 131 W; d = 0.81 [0.06 to 1.47]) ↑ P_mean CON_ (0.050 kg/m^2^)_:_ +18.6% (709 ± 146 vs. 598 ± 140 W; d = 0.80 [0.06 to 1.46]) ↑ P_mean ECC_ (0.050 kg/m^2^)_:_ +17.8% (687 ± 150 vs. 583 ± 162 W; d = 0.69 [−0.04 to 1.35]) ↑ P_mean CON_ (0.075 kg/m^2^)_:_ +21.9% (672 ± 157 vs. 551 ± 120 W; d = 0.89 [0.14 to 1.56]) ↑ P_mean ECC_ (0.075 kg/m^2^)_:_ +22.2% (709 ± 177 vs. 580 ± 145 W; d = 0.82 [0.08 to 1.48]) ↑ P_mean CON_ (0.100 kg/m^2^)_:_ +21.7% (600 ± 127 vs. 493 ± 120 W; d = 0.89 [0.14 to 1.56]) ↑ P_mean ECC_ (0.010 kg/m^2^)_:_ +13.9% (615 ± 150 vs. 540 ± 139 W; d = 0.53 [−0.18 to 1.20])
Tillin et al. [66]	17 male recreationally active athletes	2.5 h prior to exercise and 7 d of BR supplementation (NO_3_^−^ 12.9 mmol per day)	10 × MIVC leg extensions, 1 min rest	↔ Fmax: +0.27% (741 ± 136 vs. 739 ± 135 N; d = 0.02 [−0.68 to 0.71])
Williams et al. [46]	11 resistance-trained male athletes	2 h prior to exercise of BR ingestion (NO_3_^−^ 6.4 mmol)	2 × 2 at 70% 1RM free-weight bench press, 3 min rest	↑ Pmean: +19.5% (607± 112 vs. 508 ± 118 W; d = 0.19 [−0.10 to 1.76]) ↑ Vmean: +6.5% (0.66 ± 0.08 vs. 0.62 ± 0.08 m/s; d = 0.52 [−0.42 to 1.38])
Kramer et al. [67]	12 trained male CrossFit athletes	6 d of KNO_3_ supplementation (NO_3_^−^ 8 mmol per day)	2 sets × 5 isometric knee extensions/flexions, 60° flexion, 5 s rest, interspersed with 1 min rest 2 × 5 isokinetic knee extensions and flexions at 60°/s and 180°/s, 1 min rest	↔ Tpeak (isometric extension): KNO_3_ +10.2% (186 ± 49 vs. 169 ± 37 N; d = 0.42 [−0.48 to 1.23]) and PL +6.1% (185 ± 43 vs. 174 ± 28 N; d = 0.31 [−0.56 to 1.14]) ↔ Tpeak (isometric flexion): KNO_3_ +1.8% (119 ± 27 vs. 117 ± 21 N; d = 0.09 [−0.77 to 0.93]) and PL +4.8% (126 ± 20 vs. 120 ± 17 N; d = 0.33 [−0.54 to 1.16]). ↔ Tpeak (extension at 60°/s): KNO_3_ −4.1% (168 ± 50 vs. 175 ± 41 N; d = 0.16 [−1.00 to 0.70]) and PL −2.7% (179 ± 44 vs. 184 ± 48.53 N; d = 0.11 [−0.95 to 0.74]) ↔ Tpeak (flexion at 60°/s): KNO_3_ −1.5% (102 ± 26 vs. 104 ± 21 N; d = 0.07 [−0.93 to 0.77]) and PL −2.7% (104 ± 25 vs. 106 ± 25 N; d = 0.12 [−0.92 to 0.77]) ↔ Tpeak (extension at 180°/s): KNO_3_ +6.5% (128 ± 32 vs. 120 ± 36 N; d = 0.24 [−0.62 to 1.08]) and PL +2.6% (123 ± 35 vs. 120 ± 42 N; d = 0.09 [−0.77 to 0.92]) ↔ Tpeak (flexion at 180°/s): KNO_3_ +0.4% (80 ± 16 vs. 79 ± 14 N; d = 0.02 [−0.78 to 0.91]) and PL +0.8% (76 ± 20 vs. 76 ± 28 N; d = 0.02 [−0.85 to 0.85])
Jonvik et al. [40]	14 male recreationally active athletes	3 h prior to exercise and 6 d of BR supplementation (NO_3_^−^ 15.8 mmol per day)	5 × CMJ, 1 min rest 5 isokinetic knee extensions and flexions at 60°/s, 120°/s, 180°/s, and 300°/s. 3 × 4 s MIVC leg extension with 30° and 60° of flexion, 1 min rest	↔ Pmax (extension at 60°/s): +0.9% (220 ± 45 vs. 218 ± 40 W; d = 0.05 [−0.73 to 0.82]) ↔ Pmax (extension at 120°/s): +1.3% (392 ± 74 vs. 387 ± 62 W; d = 0.08 [−0.71 to 0.85]) ↔ Pmax (extension at 180°/s): +2.7% (500 ± 86 vs. 487 ± 67 W; d = 0.18 [−0.61 to 0.94]) ↔ Pmax (extension at 300°/s): +1.8% (554 ± 102 vs. 544 ± 81 W; d = 0.11 [−0.67 to 0.88]) ↑ Pmax (flexion at 60°/s): +2.0% (151 vs. 148 W; d = unknown) ↔ Pmax (flexion at 120°/s): +1.3% (392 ± 74 vs. 387 ± 62 W; d = 0.08 [−0.71 to 0.85]) ↔ Pmax (flexion at 180°/s): +2.9% (391 ± 57 vs. 380 ± 58 W; d = 0.20 [−0.59 to 0.96]) ↔ Pmax (flexion at 300°/s): +1.6% (493 ± 73 vs. 485 ± 81 W; d = 0.11 [−0.68 to 0.88]) ↔ Smax (flexion of 30°): +2.0% (204 ± 39 vs. 200 ± 37 Nm; d = 0.11 [−0.68 to 0.88]) ↔ Smax (flexion of 60°): +0.4% (286 ± 43 vs. 285 ± 47 Nm; d = 0.02 [−0.76 to 0.80]) ↔ CMJ height: −0.7% (39.3 ± 6.3 vs. 39.6 ± 6.3 cm; d = 0.05 [−0.82 to 0.73]) ↔ GRFmax: −0.5% (3.04 vs. 3.06 N; d = unknown)
Coggan et al. [37]	12 active athletes (female, *n* = 5, male, *n* = 7)	2 h prior to exercise acute BR ingestion (NO_3_^−^ 11.2 mmol)	3–4 isokinetic knee extensions at 0 rad/s, 1.57 rad/s, 3.14 rad/s, 4.17 rad/s, and 6.28 rad/s, 2 min rest	↔ P_peak_ (extension at 1.57 rad/s): −2.1% (3.31 ± 0.16 vs. 3.38 ± 0.21 W/; d = 0.39 [−1.22 to 0.49]) ↔ P_peak_ (extension at 3.14 rad/s): −1.9% (5.38 ± 0.32 vs. 5.48 ± 0.38 W/kg; d = 0.30 [−1.13 to 0.58]) ↔ P_peak_ (extension at 4.17 rad/s): +0.0% (6.67 ± 0.46 vs. 6.67 ± 0.50 W/kg; d = 0.00 [−0.85 to 0.85]) ↑ P_peak_ (extension at 6.28 rad/s): +4.1% (7.64 ± 0.52 vs. 7.34 ± 0.54 W/kg; d = 0.59 [−0.32 to 1.41]) ↔ T_peak_ (extension at 1.57 rad/s): −1.9% (2.11 ± 0.10 vs. 2.15 ± 0.11 Nm/kg; d = 0.40 [−1.22 to 0.49]) ↔ T_peak_ (extension at 3.14 rad/s): −1.8% (1.71 ± 0.10 vs. 1.74 ± 0.12 Nm/kg; d = 0.28 [−1.11 to 0.59]) ↔ T_peak_ (extension at 4.17 rad/s): +0.0% (1.42 ± 0.10 vs. 1.42 ± 0.11 Nm/kg; d = 0.00 [−0.85 to 0.85]) ↔ T_peak_ (extension at 6.28 rad/s): +4.3% (1.22 ± 0.08 vs. 1.17 ± 0.08 Nm/kg; d = 0.65 [−0.26 to 1.47]) ↔ T_max_ (0 rad/s): −1.5% (2.6 ± 0.13 vs. 2.64 ± 0.13 Nm/kg; d = 0.32 [−1.15 to 0.55])
Kokkinoplitis et al. [56]	7 healthy males	3 h prior to exercise acute BR ingestion (NO_3_^−^ 6.45 mmol)	Isokinetic knee extension and flexion at 60°/s and 240°/s	↔ T_peak_ (extension at 60°/s): −2.6% (200.2 ± 25.8 vs. 207.4 ± 37.5 Nm; d = 0.24 [−1.38 to 0.96]) ↔ T_peak_ (extension at 240°/s): −5.9% (124.1 ± 9.2 vs. 131.4 ± 17.1 Nm; d = 0.57 [−1.68 to 0.69]) ↔ T_peak_ (flexion at 60°/s): −7.4% (103.3 ± 27.7 vs. 110.9 ± 29.9 Nm; d = 0.28 [−1.42 to 0.92]) ↔ T_peak_ (flexion at 240°/s): −16.1% (59.8 ± 29.5 vs. 69.4 ± 21.5 Nm; d = 0.40 [−1.52 to 0.83])
López-Samanes et al. [57]	13 trained male tennis players	3 h prior to exercise acute BR ingestion (NO_3_^−^ 6.4 mmol)	2 MIVC handgrip 3 CMJ with 45 s of rest	↔ S_max_ (handgrip): +3.9% (47.8 ± 9.3 vs. 46.0 ± 7.9 kg; d = 0.26 [−0.61 to 1.01]) ↔ CMJ height: + 2.5% (33.0 ± 4.9 vs. 32.2 ± 5.1 cm; d = 0.143 [−0.66 to 0.97])
Haider et al. [68]	19 healthy males	2.5 h prior to exercise and 7 d of BR supplementation (NO_3_^−^ ~9.7 mmol per day)	4 × 3 s MIVC leg extension with 110° of flexion with ≥30 s rest 15 × 1 s isometric knee extensions with ≥15 s rest	↔ F_max_: (value not specified; d = unknown)

↑ = significant increase; ↓ = significant decrease; ↔ = no difference; BR = beetroot juice; CMJ = countermovement jumps; CON = concentric; ECC = eccentric; F_max_ = maximal force; GRF_max_ = peak ground reaction force; MIVC = maximal isometric voluntary contraction; *n* = sample size; NO_3_^−^ = nitrate; PL = placebo; P_max_ = maximal power; P_mean_ = mean power; P_peak_ = peak power; RM = repetition maximum; SJ = squat height; S_max_ = maximal strength; T_peak_ = peak torque, V_max_ = maximal velocity; V_mean_ = mean velocity; [ ] denotes confidence intervals.

**Table 4:** Quality assessment of studies.

Reference	Item 1	Item 2	Item 3	Item 4	Item 5	Item 6	Item 7	Item 8	Item 9	Item 10	Item 11	Item 12	Score
Buck et al. [61]	Yes	Yes	Yes	Yes	Yes	Yes	Yes	Yes	Yes	Yes	Yes	No	10/11
Rimer et al. [44]	Yes	Yes	Yes	Yes	Yes	Yes	Yes	Yes	Yes	Yes	Yes	No	10/11
Smith et al. [62]	Yes	Yes	Yes	Yes	Yes	Yes	Yes	Yes	Yes	Yes	Yes	No	10/11
Thompson et al. [60]	Yes	Yes	Yes	Yes	Yes	Yes	No	Yes	Yes	Yes	Yes	No	9/11
Wylie et al. [49]	Yes	Yes	Yes	Yes	Yes	Yes	No	Yes	Yes	Yes	Yes	No	9/11
Kent et al. [63]	No	No	Yes	No	Yes	Yes	No	Yes	Yes	Yes	Yes	No	7/11
Kokkinoplitis et al. [56]	Yes	Yes	Yes	Yes	Yes	Yes	No	Yes	Yes	Yes	Yes	No	9/11
Thompson et al. [59]	Yes	Yes	Yes	Yes	Yes	Yes	No	Yes	Yes	Yes	Yes	No	9/11
Clifford et al. [64]	Yes	Yes	Yes	Yes	Yes	Yes	Yes	Yes	Yes	Yes	Yes	No	10/11
López-Samanes et al. [57]	Yes	Yes	Yes	Yes	Yes	Yes	No	Yes	Yes	Yes	Yes	No	9/11
Ranchal-Sánchez et al. [47]	Yes	Yes	Yes	Yes	Yes	Yes	Yes	Yes	Yes	Yes	Yes	No	10/11
Rodríguez-Fernández et al. [65]	Yes	Yes	Yes	Yes	Yes	Yes	No	Yes	Yes	Yes	Yes	No	9/11
Tillin et al. [66]	Yes	Yes	Yes	Yes	Yes	Yes	Yes	Yes	Yes	Yes	Yes	No	10/11
Williams et al. [46]	Yes	Yes	Yes	Yes	Yes	Yes	Yes	Yes	Yes	Yes	Yes	No	10/11
Kramer et al. [67]	Yes	Yes	Yes	Yes	Yes	Yes	Yes	Yes	Yes	Yes	Yes	No	10/11
Jonvik et al. [40]	Yes	Yes	Yes	Yes	Yes	Yes	Yes	Yes	Yes	Yes	Yes	No	10/11
Coggan et al. [37]	Yes	Yes	Yes	Yes	Yes	Yes	No	Yes	Yes	Yes	Yes	No	9/11
Haider et al. [68]	Yes	Yes	Yes	Yes	Yes	Yes	Yes	Yes	Yes	Yes	Yes	No	10/11

## Data Availability

All details can be requested from the corresponding author.
